# Population pharmacokinetics of colistin sulfate in critically ill patients based on NONMEM

**DOI:** 10.1038/s41598-025-03503-9

**Published:** 2025-05-26

**Authors:** Qiang Sun, Xiaojing Li, Genzhu Wang, Xiaoying Wang, Baiqian Xing, Zhikun Xun, Nianfang Lu, Zhongdong Li

**Affiliations:** 1 Department of Pharmacy, Beijing Electric Power Hospital of State Grid Co. of China, Capital Medical University Electric Teaching Hospital, Beijing, China; 2 ICU, Beijing Electric Power Hospital of State Grid Co. of China, Capital Medical University Electric Teaching Hospital, Beijing, China

**Keywords:** Colistin sulfate, Population pharmacokinetics, Therapeutic drug monitoring, Dosing regimen, Two-compartment models, Drug development, Bacterial infection

## Abstract

As the last defense against multidrug-resistant gram-negative bacteria, colistin sulfate’s clinical use, which is often empirical, risks resistance and adverse reactions. This study aimed to develop a population pharmacokinetic (PPK) model of colistin sulfate for critically ill patients and determine the optimal dosing regimen. This retrospective study included 204 critically ill patients. We used a validated LC-MS/MS method to measure its plasma concentrations and RIFLE criteria for nephrotoxicity evaluation. NONMEM developed PPK models. Monte Carlo simulations set dosing regimens based on the probability of target attainment (PTA). A two-compartment model adequately described the data, creatinine clearance and weight were covariates for elimination rate and central volume, respectively. Only 11.8% had nephrotoxicity. With Monte Carlo simulations, all regimens except the maintenance dose of 0.5 MU administered every 12 h achieved > 90% PTA at the minimum inhibitory concentration (MIC) ≤ 0.5 mg/L. However, at MIC > 0.5 mg/L, the routine regimen resulted in insufficient exposure. Based on our PPK model, the dose of intravenous colistin sulfate should be adjusted according to creatinine clearance (CrCL) and weight. For critically ill patients with infections, under the conventional treatment regimens, when the MIC is ≥ 1 mg/L, it is difficult for patients to achieve the ideal therapeutic effect in terms of exposure dose. When CrCL is below 10 ml/min, the regimen of 1 MU every 8 h used could cause the potential for increasing nephrotoxicity risk, which is significantly concerned.

**Supplementary Information:**

The online version contains supplementary material available at 10.1038/s41598-025-03503-9.

## Introduction

In recent years, drug-resistant bacteria have increased significantly with the widespread clinical application of broad-spectrum antibiotics. Carbapenem-resistant microorganisms (CROs), particularly carbapenem-resistant *Enterobacteriaceae*, carbapenem-resistant *Acinetobacter baumannii*, and carbapenem-resistant *Pseudomonas aeruginosa*, are common drug-resistant bacteria in China, and the incidence of infections caused by these CROs has been gradually increasing in recent years^1^. As the final line of defense against CRO gram-negative bacterial infections^2^, three injectable types of polymyxins are used in clinical treatment, namely polymyxin B sulfate, colistin sulfate, and colistin methanesulfonate sodium (CMS). In the past decade, several studies had proved that significant individual variability in polymyxin plasma concentration occurred, particularly in critically ill patients^3–6^. Additionally, because of large inter-individual and intra-individual differences in the pharmacokinetics (PK) of most drugs due to the pathophysiological changes in critically ill patients and treatment with drug combination therapy, the guidelines^2^ recommend that (1) antibiotics should be administered based on pharmacokinetics/pharmacodynamics (PK/PD) principles and (2) the dosing regimens should be optimized to maximize clinical efficacy and reduce toxicity and drug resistance. Hence, studies on the PK of polymyxins are critical to optimize their therapeutic regimens for critically ill patients.

In 2019, the National Medical Products Administration approved the use of colistin sulfate for treating critical infections caused by gram-negative bacilli (approval no. H31020822). Thus far, most research studies were based on CMS because of limited data on the PK of colistin sulfate in patients^7–12^. Presently, CMS and colistin sulfate are thought to exhibit different PK profiles in patients^8^. A previous study based on NONMEM showed a similar pharmacokinetic profile of both colistin sulfate and polymyxin B^13^. Yu et al.^13^ developed population pharmacokinetic (PPK) model of colistin sulfate and identified a one-compartment model with first-order elimination and creatinine clearance (CrCL) as a covariate for the clearance (CL) of colistin sulfate. In another study, Xie et al.^14^ used the Phoenix NLME software and suggested a two-compartment model with first-order elimination for the CL of colistin sulfate. CrCL and alanine aminotransferase level were also investigated as covariates for CL and volume of peripheral compartmental distribution (V2), respectively. Therefore, further studies are required to understand the pharmacokinetic profile of colistin sulfate among different patients and changes in the administration doses of colistin sulfate for patients from diverse populations.

Based on the above mentioned findings, further clinical studies on the PK/PD of colistin sulfate in critically ill patients are crucial to optimize its clinical applications. Hence, in the present study, plasma samples were collected from patients receiving intravenous colistin sulfate in our hospital, and PPK models were constructed based on the scattered data. The findings of our study will provide a scientific basis for treating patients with appropriate clinical doses of colistin sulfate.

## Results

### Baseline characteristics of patients

The training set included 364 values of plasma colistin sulfate concentrations from 178 hospitalized adult patients; the 57 plasma colistin sulfate concentrations of 26 new patients were used for external validation (shown in Supplementary Table S1). Table 1 summarizes the demographic data of the patients in the training set, including information regarding basic health details, diagnosis, identified microbial pathogen and drug treatment. Most of the patients were male individuals (65.73%). Overall, 93.26% of the patients had a respiratory tract infection. Among the isolated CRO, *K. pneumoniae* had the highest proportion (51.69%), followed by *A. baumannii* (39.32%), *P. aeruginosa* (8.43%), and *Enterobacter cloacae* (3.93%).

All patients were administered colistin sulfate intravenously. The treatment duration was 11.21 ± 6.39 days. In the combination therapy, meropenem was combined most frequently with colistin sulfate (61.80%), followed by tigecycline (24.72%), fosfomycin (10.67%), and ceftazidime–avibactam (9.55%). The 14-day mortality rates of patients receiving colistin sulfate was 27.53%. 11.8% patients received colistin sulfate treatment accrued acute kidney injury.

**Table 1:** Clinical characteristics of patients.

Characteristics	Value^a^	Characteristics	Value^a^
Patient characteristics		Infection site	
Age (years)	78.5 [31, 98]	Pulmonary	166 (93.26%)
Gender (male/female)	117/61	Bloodstream	6 (3.37%)
Weight (kg)	70 [40,100]	Urinary tract	18 (10.11%)
Daily dose (MU)	1.5 [1.0, 4.0]	Abdomen	5 (2.81%)
Laboratory test results		Endocardium	1 (0.56%)
Estimated CrCL (mL/min)^b^	51.9 [6.7, 271.8]	Pathogenic bacteria	
Base line Scr (µmol/L)	79 [19, 753]	*K. pneumoniae*	92 (51.69%)
UE (mmol/L)	16.66 [2.14, 47.79]	*A. baumannii*	70 (39.32%)
ALT (U/L)	27 [3, 1133]	*P. aeruginosa*	15 (8.43%)
AST (U/L)	35 [7, 953]	*E. coli*	7 (3.93%)
ALB (g/L)	30.97 ± 4.43	Empirical	24 (13.48%)
Outcome		Combination	
C_ss.avg_ (mg/L) ^*c*^	0.93 ± 0.35	Meropenem	110 (61.80%)
14-day mortality rate	49 (27.53%)	Fosfomycin	19 (10.67%)
Acute kidney injury	21 (11.8%)	Cefoperazone/sulbactam	17 (9.55%)
hospital stay (days)	11.21 ± 6.39	Tigecycline	44 (24.72%)

^a^ Values are expressed as no. (%) or median [min, max] or mean ± SD.

^b^ CrCL was estimated using the Cockcroft-Gault equation.

^c^C_ss,avg_, the steady-state plasma concentration of surviving patients.

^d^ CrCL, creatinine clearance estimated by the Cockcroft-Gault equation; Scr, serum creatinine; ALT, alanine transaminase; AST, aspartic transaminase; ALB, albumin; PT, prothrombin time.

### PPK analysis

PPK modeling was performed for 178 patients with 364 values of colistin sulfate concentrations ranging from 0.16 to 4.91 mg/L. First-dose peak concentrations were achieved in 52 concentrations (14.29%), whereas peak and trough concentrations were noted in 154 concentrations (42.31%) and 158 concentrations (43.41%), respectively. Two-compartment models with linear elimination showed better performance than one-compartment models (objective function value (OFV): −233.704 vs. −191.872). Therefore, two-compartment models with first-order elimination were selected as the base models. Residual random effects were assessed using additive plus proportional error models. We established the covariate model using the stepwise method involving forward inclusion and backward elimination. The final results indicated that age, sex, albumin, and other clinical variables had no statistically significant correlation with the pharmacokinetic (PK) parameters. The final model identified CrCL as an effective covariate for the central compartment CL and Weight (WT) as an effective covariate for the apparent volume of the central compartment distribution (V1). The final PPK model is shown in Eqs. 1–4. Table 2 shows the parameter values of PPK assessment and bootstrap replicates validation results. As shown in Supplementary Figure S1, prediction-corrected visual predictive check (pc-VPC) showed better stability of the final model.

1 $$\:{\text{CL(L/h) = 2}}{\text{66}}{\left( {\frac{{{\text{CrCL}}}}{{{\text{71}}{\text{.40}}}}} \right)^{{\text{0}}{\text{.456}}}}{\text{exp}}\left( {\eta {\text{CL}}} \right)$$

2 $$\:{\text{V1(L) = 49}}{\text{.70 }}{\left( {\frac{{{\text{WT}}}}{{{\text{67}}{\text{.89}}}}} \right)^{{\text{1}}{\text{.2}}}}{\text{ exp(}}\eta {\text{V1)}}$$

3 $$\:{\text{Q(L/h) = }}{\text{1.63exp(}}\eta {\text{Q)}}$$

4 $$\:{\text{V2(L) = 109 exp(}}\eta {\text{V2)}}$$

**Table 2:** Parameter estimates and bootstrap results of the final PPK model.

Parameter	Final model				Bootstrap	
	Estimate	RSE (%)	95% CI	Shrinkage (%)	Median	95% CI
tvV1(L)	49.7	5.8	44.036–55.364	NA	50.04	44.16–55.26
tvV2(L)	109	24.8	56.08–161.92	NA	103.65	58.18–161.31
tvCL (L/h)	2.66	7	2.293–3.027	NA	2.67	2.30–3.04
tvQ (L/h)	1.63	19.8	0.999–2.261	NA	1.67	0.44–3.67
dCLdCrCL	0.456	8.6	0.379–0.533	NA	0.466	0.39–0.59
dVdWT	1.2	22.2	0.679–1.721	NA	1.28	0.60–1.75
Interindividual variability						
ω^2^V1	0.244	19	NA	21.9	0.215	0.16–0.34
ω^2^CL	0.125	22	NA	38.5	0.117	0.05–0.19
ω^2^Q	1.69	49	NA	60.7	1.657	0.12–6.00
ω^2^V2	0.993	29	NA	57.6	0.892	0.10–4.00
Residual variability(σ)						
stdev0	1			*40.8*		

RSE%, relative standard error; CI, confidence interval; tvV1, typical value of volume of central compartment distribution (V1); tvV2, typical value of volume of peripheral compartment distribution (V2); tvCL, typical value of central compartment clearance (CL); tvQ, typical value of inter-compartmental clearance (Q); dCLdCrCL, exponential parameter coefficient of creatinine clearance (CrCL) to CL; dV1 dWT, exponential parameter coefficient of WT to V1; ω^2^V1, variance of inter-individual variability for V1; ω^2^CL, variance of inter-individual variability for CL; ω^2^V2, variance of inter-individual variability for V2; ω^2^Q, variance of inter-individual variability for Q; stdev0, standard deviation.

**Fig. 1:**
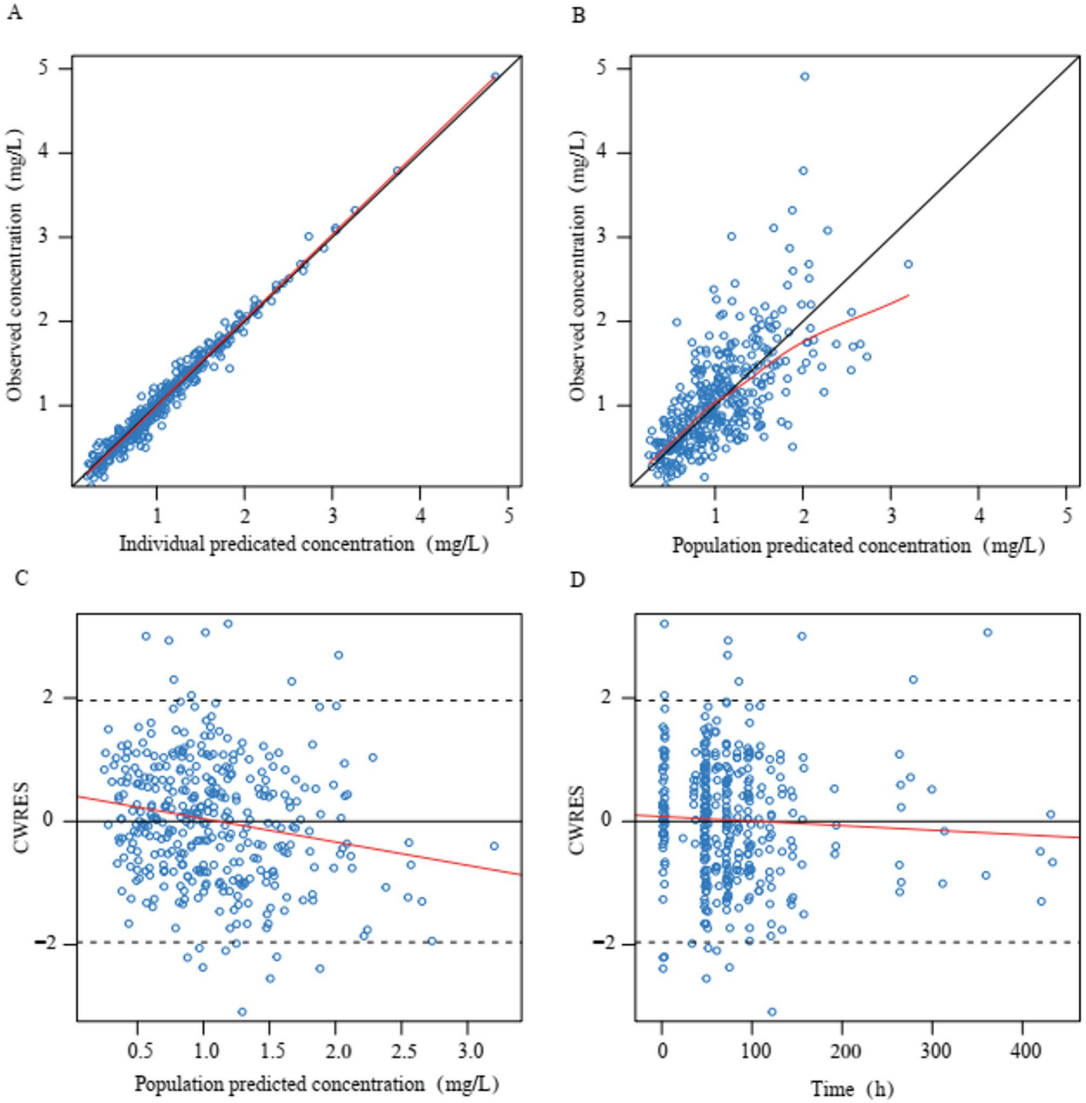
Diagnostic goodness-of-fit plots of the final model. (**A**) Observed concentration (DV) vs. individual predicted concentration (IPRED); (**B**) DV vs. population predicted concentration (PRED); (**C**) conditional weighted residuals (CWRES) vs. PRED; and (**D**) CWRES vs. time. The red lines in the upper panel represent loess smooth lines and linear fit lines, respectively.

The observation data of goodness-of-fit plots for the final model are shown in Fig. 1. A close correlation was noted between the observed concentrations and population predicted concentration (PRED) and individual predicted concentration (IPRED) in the scatterplots. The conditional weighted residual (CWRES) with time and PRED of the final model revealed a randomly normal distribution, and most residuals were focused on zero within an accepted range (− 2 to 2). The 5 th, 50 th, and 95 th percentiles of the simulated concentrations based on the observation and simulation data were within the 90% confidence intervals (CIs) of the corresponding quantiles. As shown in Supplementary Figure S1, pc-VPC revealed that the final model could reliably predict the observation data. Overall, the results supported the predictive performance of the final model and its application for subsequent dosing simulations.

A total of 57 concentrations from 26 new patients who met the inclusion and exclusion criteria were considered the test dataset. Supplementary Table S1 shows the details of these 26 patients. There were no significant differences in WT and CrCL between these 26 patients and the main cohort (WT: *U* = 2017.50, *P* = 0.288; CrCL: *U* = 1995.50, *P* = 0.257). By combining the final model with the WT and CrCL of these patients, the plasma colistin sulfate concentrations were estimated (Supplementary Table S2). The plots of external validation prediction capability are shown in Supplementary Table S3. The mean squared error (MSE), mean absolute error (MAE), root-mean-square error (RMSE) and mean absolute percentage error (MAPE) results demonstrated that the final model could accurately predict the plasma colistin sulfate concentration of these patients; moreover, colistin sulfate peak concentration values were considerably more accurately predicted, and the predictive performance of the final model was reliable.

### Monte Carlo simulations results

**Fig. 2:**
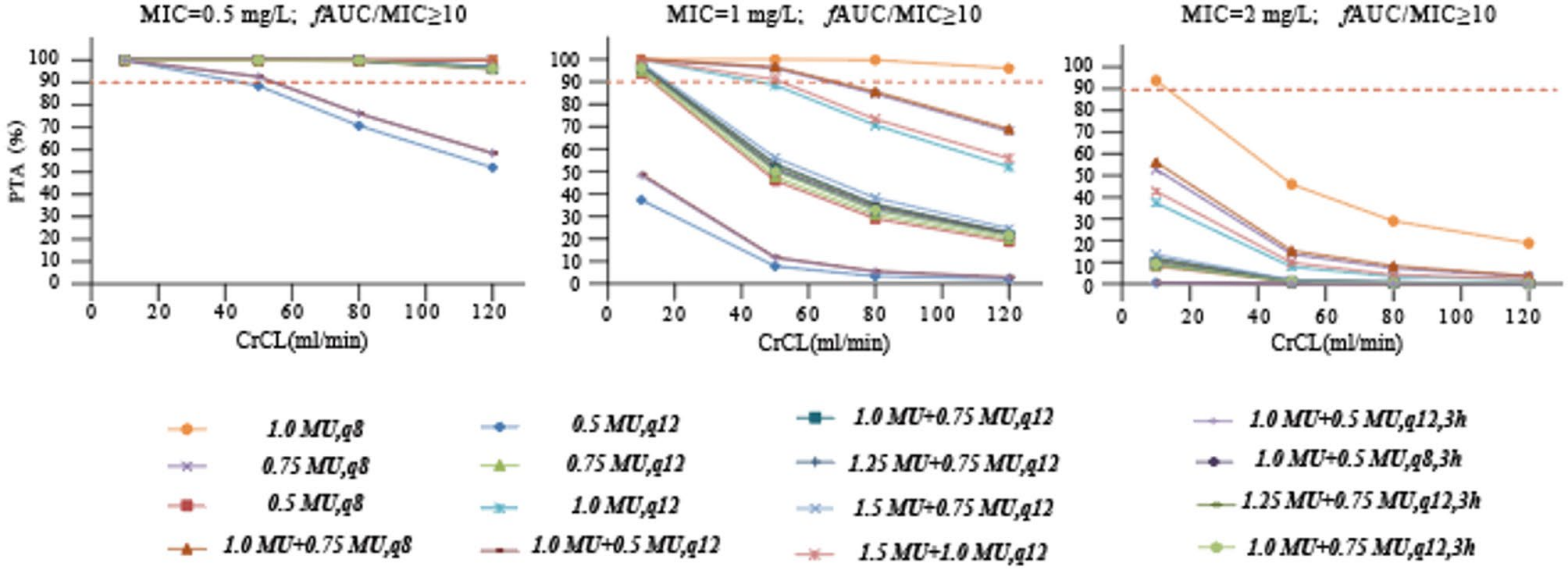
Simulated probability of achieving target attainment (***f***AUC/MIC ≥ 10) of colistin sulfate at each MIC (mg/L) for different dose regimens in patients with various CrCL volumes. The dashed horizontal line indicates 90% PTA. CrCL, creatinine clearance; PTA, probability of target attainment. In the last four dosing regimens, 3 h indicates an infusion time of three hours.

The probability of target attainment (PTA) for different dose regimens of colistin sulfate at each minimum inhibitory concentration (MIC) is presented in Fig. 2. For patients with various CrCL levels, when the MIC = 0.5 mg/L, only the PTA of the maintenance dose regimens of 0.5 MU administered every 12 h were less than 90%. However, when the MIC exceeded 1 mg/L, the PTA at the recommended dosage, as specified in the instruction manual, was below 90% for all cases. This suggested that a dosage higher than that recommended in the instruction manual was necessary to achieve the desired target attainment probability for elevated MIC values. Additionally, for MIC = 2 mg/L, all the simulated dose regimens failed to achieve a PTA ≥ 90%. Moreover, with the same dose, the 8-hour dosing interval had a higher PTA achievement compared to the 12-hour dosing interval.

**Fig. 3:**
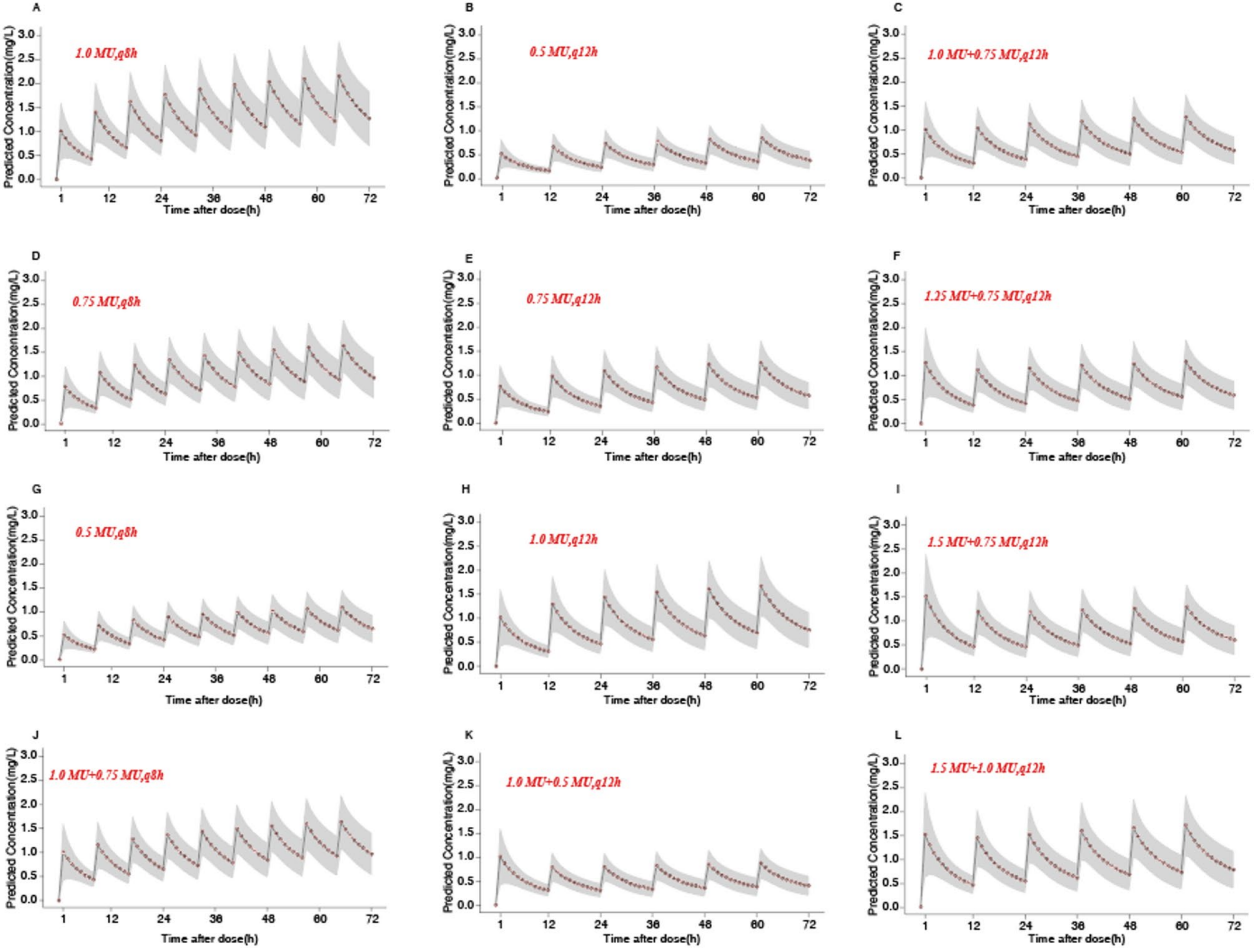
Concentration–time profiles of colistin sulfate. The dots represent the average predicted concentration for each hour; the black line represents the predicted drug time curve; the shaded area represents the range of predicted concentrations.

The concentration–time profiles of colistin sulfate with different dosing regimens are shown in Fig. 3. The results revealed that the concentration–time profiles of colistin sulfate varied for patients who received intravenous colistin sulfate with different dosing regimens. According to the overall trend, the greater the drug dose, the higher was the plasma colistin sulfate concentration. When the treatment plan includes a loading dose (Fig. 3C, F, I, J, K and L), the blood colistin concentrations will reach steady state more rapidly compared to those on a non-loading dose regimen (Fig. 3A, B, D, E, G and H), which indicated that the loading dose was essential for the blood colistin concentrations to quickly reach steady state. Under the same maintenance doses, the greater the loading dose, the earlier the time at which plasma colistin sulfate concentration achieved a steady state (Fig. 3F, I). Moreover, under the same loading dose, the higher the maintenance dose administered at the same time, the higher the cumulative plasma colistin sulfate concentration was (Fig. 3I, J). Regardless of whether a loading dose is given or not, the steady-state concentration of the dosing regimen of 0.5 MU every 12 h is the lowest, and it is not recommended to use this regimen in patients with normal renal function; based on the results of PTA (MIC = 0.5 mg/L), the maintenance dose should be at least 0.75 MU to achieve the desired goal (PTA > 90%).

**Fig. 4:**
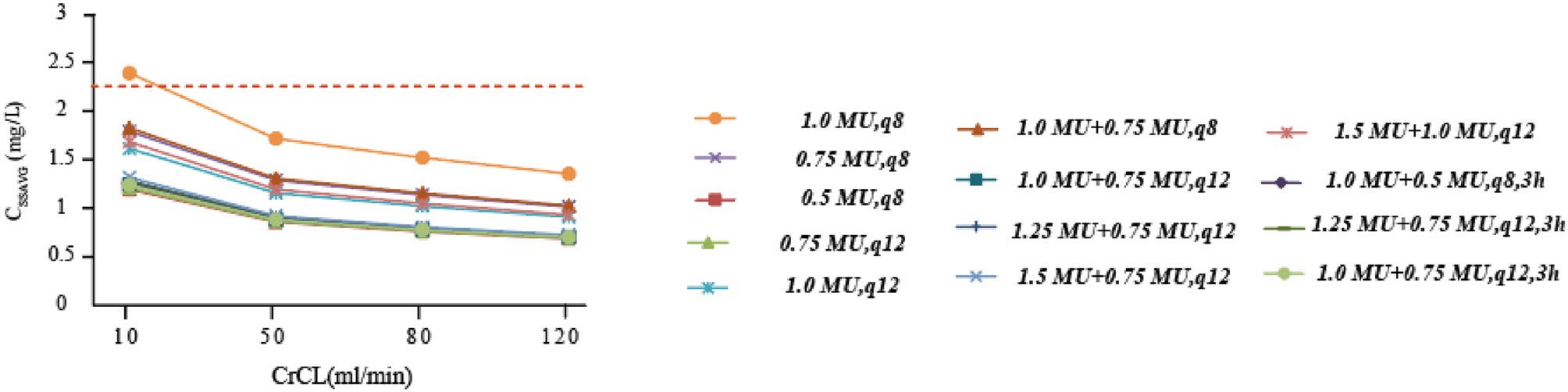
Predicting the steady-state blood drug concentration in patients. The blue dashed line represents the C_ss.avg_ of patients who experienced nephrotoxicity after empirical administration. CrCL, creatinine clearance; In the last three dosing regimens, 3 h indicates an infusion time of three hours; The red dotted line represents 2.3 mg/L.

We estimated the C_ss.avg_ for the treatment regimen with PTA exceeding 90% using Monte Carlo simulation. The results indicated that as CrCL decreased, the C_ss.avg_ in patients tended to increase gradually. Specifically, when CrCL was below 10 ml/min, the C_ss.avg_ of a regimen dosed at 1 MU every 8 h might exceeded 2.3 mg/L (Fig. 4).

## Discussion

The increase in the incidence of severe multidrug-resistant bacterial infections and the limitations of the recommended therapeutic regimens for multidrug-resistant gram-negative bacteria (MDR-GNB) infections have spurred a renewed interest in the clinical application of polymyxins as the first-line therapy for these infections^15^. Thus far, there have been limited studies on the PPK of colistin sulfate in adult critically ill patients, and the number of the included samples was smaller^13,14^. The present study included 178 critically ill patients with 364 values of plasma colistin sulfate concentrations, which were used to construct PPK models. The final model identified CrCL and WT as effective covariates for the central compartment CL and the apparent V1, respectively. The results supported the predictive performance of the final model and its application for subsequent dosing simulations.

We chose the first-order elimination double chamber model as the basic model for this study, which is the same as that used by Xie et al.^14^ in the present study. The final model identified CrCL and WT as effective covariates for the central compartment CL and the apparent V1, respectively. Previous studies on PPK in adult critically ill patients also revealed CrCL as an effective covariate for CL^13,14^, which is the same as our study. A noteworthy finding is that identified the WT as effective covariates for the apparent V1, which is inconsistent with Xie et al.^14^ in the present study. The apparent volume of the central compartment distribution in this study was approximately double that reported in previous studies^13,14^, considering factors such as the slightly higher body weight of the included population (WT 70 (40–100) kg), the larger variation in weight, the greater tissue volume^16^ and the distribution volume compared with Yu et al. (WT 63.47 ± 9.64 kg) and Xie et al. (WT 55 (45–65) kg)^13,14^, thus it might be V1 associated with the WT value noted in our present study. Our models showed a correlation between CrCL and CL, thus indicating that patients with renal insufficiency had higher exposure to colistin sulfate. Therefore, we considered that dose adjustments for colistin sulfate are recommended according to renal function.

We found that colistin sulfate at a low dose still had a positive influence on patients. Our findings showed that 129 patients (14-day mortality rate 27.53%) experienced alleviation of clinical symptoms or achieved clinical cure after receiving colistin sulfate treatment. The steady-state plasma concentration (C_ss.avg_) of these 129 patients was 0.93 ± 0.35 mg/L, which was inconsistent with the value (2.0–2.4 mg/L) jointly recommended by the multidisciplinary expert consensus^17^ on the rational clinical use of colistin in China. This consensus considered that the in vitro antimicrobial activity of polymyxin sulfate B was almost similar to that of polymyxin E; hence, the PK/PD index and target values of these two drugs might be mutually referenced. However, because the guideline was based on experiments with CMS, some studies^18^ indicated that values of colistin sulfate were significantly lower than those of CMS due to differences in their PK parameters. Additionally, in the study of Yu et al.^14^, the C_ss.avg_ of colistin sulfate in 14 patients with successful treatment was 1.73 ± 0.98 mg/L, which was also lower than that recommended by the multidisciplinary expert consensus. However, some studies have shown that in patients receiving intravenous colistin sulfate, factors such as treatment duration, ALB, APACHE II score, and use of vasoactive drugs can all affect the clinical efficacy^19^. Moreover, this study was conducted in a specific patient population, with limited sample size, no control group and no subgroup analysis performed. Therefore, whether low-level exposures are sufficient to achieve an ideal therapeutic effect still requires further exploration and verification.

In our study, among the clinical isolates of colistin resistance, there were 92 cases (51.69%) of *K. pneumoniae*, 70 cases (39.32%) of *A. baumannii*, and 15 cases (8.43%) of *P. aeruginosa* (Table 1). Given that in multiple previous studies, the MICs of polymyxin B against the afore mentioned clinical isolates were mostly 0.5 mg/L and 1 mg/L^20^. In addition, the European Committee on Antimicrobial Susceptibility Testing recommends a “susceptible” breakpoint of < 2 mg/L for polymyxins. Considering that polymyxin B and colistin exhibited basically the same antibacterial activity in vitro, the MICs selected for Monte Carlo simulation in our study were determined to be 0.5, 1, and 2 mg/L^13,14,21^. Previous in vitro and animal studies have consistently shown that the PK/PD index of polymyxins is *f*AUC/MIC. In addition, based on multiple studies of colistin sulfate E and polymyxin B, *f*AUC_0–24_/MIC ≥ 10.0 was selected as the PK/PD target^22–24^.Therefore, we finally determined it as the PK/PD target. The results showed that at the recommended dosage in the instruction manual (daily dose 1.0–1.5 MU), only when MIC = 0.5 mg/L can the target compliance rate of PTA > 90% be achieved. At higher MIC values, higher doses are required. When the MIC is higher than 2 mg/L, it is determined to be a drug-resistant strain^15^. At this time, the best treatment plan can choose combination therapy or alternative anti-infective regimens. However, our hospital did not estimate the MIC of colistin. Regarding clinical results, although the patients showed good clinical outcomes, we could not determine the influence of the *f*AUC/MIC on clinical outcomes due to the lack of drug sensitivity tests.

As reported in previous studies, the incidence rate of nephrotoxicity induced by colistin sulfate is less than 21.25%^14,25–29^, which is similar to the 11.8% observed in our study after receiving empirical administration of colistin sulfate. Advanced age, the presence of multiple coexisting diseases, severe infections, low fluid replacement volume, and exposure to combination therapy with multiple drugs are recognized as risk factors for the development of acute kidney injury associated with colistin sulfate treatment^30^. If colistin sulfate is the only factor considered, a C_ss_ level of at least 2.3 mg/L may serve as a risk factor for kidney injury^17^. In the Monte Carlo simulation, we observed that as CrCL decreased, the C_ss.avg_ of the patients we simulated tended to gradually increase. When CrCL was below 10 ml/min, the C_ss.avg_ for a treatment regimen of 1 MU every 8 h might exceed 2.3 mg/L^17^, potentially increasing the risk of nephrotoxicity associated with this drug. However, such a regimen is not commonly employed in clinical practice. If this regimen were to be adopted, it would be critical to actively monitor and manage nephrotoxicity risks, particularly in patients with CrCL values below 10 ml/min.

The present study has some limitations. Firstly, this study included a relatively large number of patients, but it was a retrospective, single-center investigation, bias could not be controlled completely during the evaluation of clinical outcomes of colistin sulfate Secondly, the data points of the first - dose peaks included in our model accounted for only 14.29%. This small proportion meant that there was insufficient information regarding the initial rapid distribution phase of the drug in the body. In the next stage of our work, we will increase the number of blood sampling points and ensure their rational distribution. Thirdly, although the simulated PK/PD prediction outcomes were very accurate, the lack of MIC determination imposed certain limitations on further implementation of the dose regimens in clinical practice. Finally, future prospective studies are required to evaluate the PPK of colistin sulfate and its clinical outcomes in a larger multiple central population. In general, our developed model showed steady and accurate prediction of plasma colistin sulfate concentration.

## Materials and methods

### Study design and patient population

The retrospective study subjects were patients admitted to Beijing Electric Power Hospital, Beijing, China, between May 2022 and May 2024. The study design conformed to the principles of the Declaration of Helsinki, and the study was approved by the Ethics Research Committee of Beijing Electric Power Hospital (approval no: n090327). Written informed consent was obtained from all patients or their legal representatives. In clinical practice, adult patients who had received intravenous colistin sulfate (Specification: 0.5 MU; Asia Pioneer Pharmaceutical Co., Ltd., Shanghai, China) with at least one measurement of colistin sulfate concentration were eligible for inclusion. The inclusion criteria were as follows: (1) age ≥ 18 years, (2) patients who had received colistin sulfate treatment, and (3) patients with at least one measurement of colistin sulfate concentration. The exclusion criteria were as follows: (1) patients who died within 24 h after receiving colistin sulfate treatment, (2) patients who received nebulized colistin sulfate and intrathecal colistin sulfate, and (3) patients who also received continuous renal replacement therapy during colistin sulfate treatment. The infection under control what needs to meet one of condition as follows: (1) patients who showed improvements in their general health conditions, infection laboratory values, serum biochemical, and imaging finding, (2) patients who received antibiotic de-escalation and (or) drug discontinuance, (3) patients who recovered successfully and discharged. The RIFLE criteria (risk, injury, failure, loss, and end-stage kidney disease), estimated with exclusion of the urinary output criterion, were used for detection and stratification of acute kidney injury^31^.

### Blood sample collection and determination of plasma colistin sulfate concentration

The members of the Antibiotics Committee decided whether to administer colistin sulfate to the patient as well as its dosing regimen (dose amount, dose interval, mode of administration, and treatment duration). The product label sheet recommended administration of colistin sulfate through an intravenous drip at the daily dose of 1.0–1.5 MU is divided into 2–3 sessions of the maintenance dose. Our hospital recommended colistin sulfate administration through an intravenous drip at the loading dose of 1.0–2.0 MU and a daily dose of 1.0–1.5 MU divided into 2–3 sessions of the maintenance dose.

Blood samples were collected from the arm opposite to the one receiving the infusion. To separate plasma, the samples were centrifuged for 10 min at 4 ℃ and 4000×g immediately after sampling. The obtained plasma was stored at −80℃. A high-performance liquid chromatography-tandem mass spectrometry platform (4500MD; AB Sciex, Framingham, MA, USA; Reg. No.: 20172221554) was used for analysis. The plasma colistin sulfate concentration was determined by a validated LC-MS/MS method from our previous study^32^. This method was validated by the validation test of Chinese Pharmacopoeia 2020 version and the US FDA guidelines for the validation of bioanalytical methods. The total concentration of colistin sulfate is calculated as the sum of colistin A and colistin B. The detection concentration ranges of colistin A and colistin B by this method are 0.06–4.00 mg/L and 0.10–7.00 mg/L, respectively^32^.

### Clinical data collection

The precise time of colistin sulfate administration and blood sample collection was retrieved from the computerized medical records. The following details were also collected from the hospital medical record system: patient demographic characteristics, clinical diagnoses, infection sites, pathogenic bacteria, laboratory test results, and information on administered drugs and drug combination therapy. CrCL was estimated using the Cockcroft-Gault equation (Eq. 5); for females, the obtained results were multiplied by 0.85.

5 $$\:\text{C}\text{r}\text{C}\text{L}\,(\text{m}\text{L}/\text{m}\text{i}\text{n}\:)=\frac{\left(140-\text{A}\text{G}\text{E}\right)\times\:\text{W}\text{T}\left(\text{k}\text{g}\right)}{0.818\times\:Scr({\upmu\:}\text{m}\text{o}\text{l}/\text{L})}$$

### PPK models of colistin sulfate

PPK analyses were performed using NONMEM version 7.5 (Icon Development Solutions, Ellicott City, MD, USA) and Pirana version 3.0. Ggplot2 version 3.5.1 package in R software (version 4.1.3, R-project.org) and Xpose version 4.3.2 packages were used to generate line plots and diagnostic plots, respectively.

One- and two-compartment structural models with first-order elimination characteristics of colistin sulfate were constructed using NONMEM software. Between-subject variability (BSV) was assessed using an exponential function. Within-subject variability (WSV) was assessed by additive, proportional, or combined (additive plus proportional) models, while model with the smallest variability represented the best ones. The base model was chosen according to diagnostic plots and goodness-of-fit criteria, including improvement of the OFV and precision and plausibility of parameter estimation. The factors influencing fixed effects were screened based on our previous study^32^. Covariates were included in the base models in a stepwise manner, and testing was performed by using the following statistical criteria: ΔOFV > 3.84, *P* < 0.05. The covariate was considered to have a significant influence at ΔOFV > 3.84 and retained in the model or eliminated otherwise. All covariates with significant influence on models were then incorporated into the base models to construct full regression models. The full regression models were then subjected to reverse elimination. The included covariates were tested using stricter statistical criteria (ΔOFV > 6.63, *P* < 0.01), and covariates showing a small influence on the models were eliminated, thereby yielding the final model.

Goodness-of-fit plots, nonparametric bootstrap, and pc-VPC were used to evaluate the final model and parameter estimates. A nonparametric bootstrap procedure was utilized to assess the performance and stability of the final model. Random sampling and individual replacement were used to generate 1000 replicate datasets. The median and 95% CI values of the final model parameters obtained using bootstrap validation were then compared, and the predictive performance of the model was validated. The dataset was simulated 1000 times using the $SIMULATION block in NONMEM. The 90% CI values for the 5 th, 50 th, and 95 th percentiles of the simulated concentrations were calculated. The time curves were then plotted after the last dose and compared with the observed concentrations for prediction and variability correction using pc-VPC.

Collect the clinical data and blood concentration data of colistin sulfate from patients who have used it, meet the inclusion criteria but are not included in the final model, and use these data as the test dataset. The constructed PPK final model was used to predict the plasma concentrations of these patients. The accuracy and precision of model prediction were evaluated through external validation by using MSE, MAE, RMSE and MAPE.

### Monte Carlo simulations

The final model was utilized to conduct Monte Carlo simulations on 1,000 virtual patients for the purpose of identifying the practical dose adjustment of colistin sulfate. The *f*AUC/MIC has been proven to be the PK/PD index that can best predict the bactericidal effect of colistin in the infection model of the mice thigh infection model. Based on previous retrospective studies on colistin sulfate, we finally selected 16 different dosing regimens (with a loading dose ranging from 1.0 to 1.5 MU and a maintenance dose ranging from 0.5 to 1.0 MU, etc.). Combined with different MICs (0.5 mg/L, 1 mg/L, 2 mg/L), we respectively calculated the PTA of different ratios of *f*AUC/MIC (For achieving a 2 log_10_ reduction in bacterial count, the target values of the ratio of *f*AUC/MIC were approximately 10 for both *Pseudomonas aeruginosa* and *Acinetobacter baumannii.* The unbound fraction was defined as 0.5^22^. In our study, a PTA greater than 90% was the optimal target for dose selection.

Simultaneously, the $ESTIMATION block in NONMEM was utilized to generate the plasma concentration-time curves based on the data of 1,000 simulated critically ill patients. The objective was to monitor the mean ± standard deviation values of drug concentrations in critically ill patients who were administered intravenous colistin sulfate at diverse time points and with varying dosing regimens.

### Statistical analysis

SPSS22.0 software was used for statistical analyses (IBM Corp. in Armonk, NY, USA). Data are presented as n (%), mean ± SD, or median (range). For non–normally distributed continuous variables, differences between two groups were analysed using the Mann–Whitney U test.

## Electronic supplementary material

Below is the link to the electronic supplementary material.

Supplementary Material 1

## Data Availability

The datasets generated during and/or analysed during the current study are available from the corresponding author on reasonable request.
