# Supplementary material for: Population pharmacokinetics of colistin sulfate in critically ill patients based on NONMEM
Source: Sci Rep. 2025 May 26;15:18295. doi: 10.1038/s41598-025-03503-9 (PMC12106615; doi:10.1038/s41598-025-03503-9)
Supplement: Supplementary file 1 — Supplementary Material 1 [file 41598_2025_3503_MOESM1_ESM.docx]

**Supplementary Table 1** Characteristics of intravenous colistin sulfate external validation

| ID | Gender | Age (years) | Wight (kg) | Daily dose | Estimated Ccr(mL/min) | Infection site | Pathogenic bacteria |
| --- | --- | --- | --- | --- | --- | --- | --- |
| 1 | F | 60 | 70 | 100IU,q12h | 44.08 | Pulmonary | *P. aeruginosa* |
| 2 | M | 97 | 65 | 125+75IU,q12h | 64.47 | Pulmonary | *A. baumannii* |
| 3 | M | 68 | 80 | 100IU,q12h | 138.07 | Pulmonary | *K. pneumoniae* |
| 4 | M | 45 | 90 | 100IU,q12h | 13.96 | Pulmonary | *K. pneumoniae* |
| 5 | F | 72 | 60 | 100IU,q12h | 100.94 | Pulmonary | *K. pneumoniae* |
| 6 | M | 73 | 55 | 125+75IU,q12h | 63.45 | Pulmonary | *None* |
| 7 | M | 93 | 60 | 150+100IU,q12h | 23.78 | Pulmonary | *A.baumannii, K. pneumoniae* |
| 8 | M | 87 | 55 | 100IU,q12h | 60.40 | Pulmonary | *A. baumannii* |
| 9 | F | 78 | 55 | 100IU,q12h | 101.24 | Pulmonary | *A. baumannii* |
| 10 | M | 71 | 60 | 100IU,q12h | 99.24 | Pulmonary, Urinary tract | *K. pneumoniae* |
| 11 | M | 86 | 70 | 125+75IU,q12h | 44.86 | Pulmonary | *None* |
| 12 | M | 94 | 80 | 150+100IU,q12h | 65.2 | Pulmonary | *A. baumannii* |
| 13 | F | 88 | 60 | 150+100IU,q12h | 8.62 | Pulmonary | *A. baumannii, K. pneumoniae* |
| 14 | M | 79 | 73 | 125+75IU,q12h | 106.74 | Pulmonary, Urinary tract | *K. pneumoniae* |
| 15 | M | 92 | 65 | 150+100IU,q12h | 41.91 | Pulmonary | *K. pneumoniae* |
| 16 | M | 75 | 68 | 100IU,q12h | 96.49 | Pulmonary, Abdomen | *K. pneumoniae* |
| 17 | M | 89 | 68 | 150+100IU,q12h | 16.24 | Pulmonary, Bloodstream | *K. pneumoniae* |
| 18 | M | 77 | 65 | 100IU,q12h | 192.54 | Pulmonary | *A. baumannii* |
| 19 | M | 48 | 58 | 150+100IU,q12h | 163.08 | Pulmonary | *A. baumannii, K. pneumoniae* |
| 20 | F | 85 | 55 | 100IU,q12h | 38.81 | Pulmonary, Bloodstream | *A. baumannii* |
| 21 | F | 90 | 57 | 125+75IU,q12h | 75.94 | Pulmonary | *None* |
| 22 | M | 64 | 70 | 100IU,q12h | 162.59 | Pulmonary | *A. baumannii* |
| 23 | M | 58 | 64.8 | 125+75IU,q12h | 129.92 | Pulmonary | *A. baumannii* |
| 24 | M | 95 | 65 | 100IU,q12h | 53.37 | Pulmonary | *A. baumannii* |
| 25 | F | 60 | 70 | 100IU,q12h | 17.01 | Pulmonary, Urinary tract | *A. baumannii* |
| 26 | M | 59 | 65 | 150+100IU,q12h | 143.03 | Pulmonary | *A. baumannii* |

**Supplementary Table 2 External validation and error table**

| **No.** | **Administered dose** | **Blood collection time point ^a^** | **concentration (mg/L)** | **Prediction value(mg/L)** | **Error value (%)** |
| --- | --- | --- | --- | --- | --- |
| *1* | Initial dose 100 IU; Each dose 100 IU，Q12h;  Infusion time 1h | *First dose peak concentration* | NA | NA | NA |
|  |  | *Trough concentration（5th dose）* | 1.09 | 0.95 | 13 |
|  |  | *Peak concentration（5th dose）* | 1.43 | 1.52 | 6 |
| *2* | Initial dose 125 IU;  Each dose 75 IU，Q12h;  Infusion time 3h | *First dose peak concentration* | 0.93 | 0.94 | 1 |
|  |  | *Trough concentration（5th dose）* | 0.82 | 0.75 | 9 |
|  |  | *Peak concentration（5th dose）* | 1.10 | 1.12 | 2 |
| *3* | Initial dose 100 IU;  Each dose 100 IU，Q12h;  Infusion time 1h | *First dose peak concentration* | NA | NA | NA |
|  |  | *Trough concentration（6th dose）* | 0.38 | 0.43 | 14 |
|  |  | *Peak concentration（6th dose）* | 1.22 | 1.21 | 1 |
| *4* | Initial dose 100 IU; Each dose 100 IU，Q12h;  Infusion time 1h | *First dose peak concentration* | NA | NA | NA |
|  |  | *Trough concentration（10th dose）* | 1.64 | 1.60 | 2 |
|  |  | *Peak concentration（10th dose）* | 2.12 | 2.14 | 1 |
| *5* | Initial dose 100 IU; Each dose 100 IU，Q12h;  Infusion time 1h | *First dose peak concentration* | NA | NA | NA |
|  |  | *Trough concentration（5th dose）* | 0.36 | 0.39 | 11 |
|  |  | *Peak concentration（5th dose）* | 1.01 | 1.09 | 8 |
| *6* | Initial dose 125 IU; Each dose 75 IU，Q12h;  Infusion time 3h | *First dose peak concentration* | 0.74 | 0.81 | 9 |
|  |  | *Trough concentration（5th dose）* | 0.63 | 0.60 | 6 |
|  |  | *Peak concentration（5th dose）* | 0.96 | 0.97 | 2 |
| *7* | Initial dose 150 IU; Each dose 100 IU，Q12h;  Infusion time 1h | *First dose peak concentration* | 0.41 | 0.63 | 52 |
|  |  | *Trough concentration（11th dose）* | 1.58 | 1.57 | 1 |
|  |  | *Peak concentration（11th dose）* | 2.00 | 1.96 | 2 |
| *8* | Initial dose 100 IU; Each dose 100 IU，Q12h;  Infusion time 1h | *First dose peak concentration* | NA | NA | NA |
|  |  | *Trough concentration（7th dose）* | 0.82 | 0.75 | 8 |
|  |  | *Peak concentration（7th dose）* | 1.37 | 1.44 | 5 |
| *9* | Initial dose 100 IU; Each dose 100 IU，Q12h;  Infusion time 1h | *First dose peak concentration* | NA | NA | NA |
|  |  | *Trough concentration（5th dose）* | 0.78 | 0.7 | 11 |
|  |  | *Peak concentration（5th dose）* | 1.49 | 1.51 | 2 |
| *10* | Initial dose 100 IU; Each dose 100 IU，Q12h;  Infusion time 1h | *First dose peak concentration* | NA | NA | NA |
|  |  | *Trough concentration（5th dose）* | NA | NA | NA |
|  |  | *Peak concentration（5th dose）* | 1.60 | 1.58 | 1 |
| *11* | Initial dose 125 IU; Each dose 75 IU，Q12h;  Infusion time 3h | *First dose peak concentration* | 0.92 | 0.92 | 0 |
|  |  | *Trough concentration（5th dose）* | 0.72 | 0.69 | 3 |
|  |  | *Peak concentration（5th dose）* | 1.11 | 1.12 | 1 |
| *12* | Initial dose 150 IU; Each dose 100 IU，Q12h;  Infusion time 1h | *First dose peak concentration* | NA | NA | NA |
|  |  | *Trough concentration（12th dose）* | 0.86 | 0.88 | 2 |
|  |  | *Peak concentration（12th dose）* | 1.60 | 1.59 | 1 |
| *13* | Initial dose 150 IU; Each dose 100 IU，Q12h;  Infusion time 1h | *First dose peak concentration* | NA | NA | NA |
|  |  | *Trough concentration（5th dose）* | 1.04 | 1.02 | 1 |
|  |  | *Peak concentration（5th dose）* | 1.73 | 1.77 | 2 |
| *14* | Initial dose 125 IU; Each dose 75 IU，Q12h;  Infusion time 3h | *First dose peak concentration* | NA | NA | NA |
|  |  | *Trough concentration（11th dose）* | 0.66 | 0.65 | 1 |
|  |  | *Peak concentration（11th dose）* | 1.35 | 1.30 | 4 |
| *15* | Initial dose 150 IU; Each dose 100 IU，Q12h;  Infusion time 1h | *First dose peak concentration* | NA | NA | NA |
|  |  | *Trough concentration（16th dose）* | 1.30 | 1.13 | 13 |
|  |  | *Peak concentration（16th dose）* | 1.38 | 1.57 | 14 |
| *16* | Initial dose 100 IU; Each dose 100 IU，Q12h;  Infusion time 1h | *First dose peak concentration* | NA | NA | NA |
|  |  | *Trough concentration（11th dose）* | 0.43 | 0.50 | 15 |
|  |  | *Peak concentration（11th dose）* | 1.45 | 1.45 | 0 |
| *17* | Initial dose 150 IU; Each dose 100 IU，Q12h;  Infusion time 1h | *First dose peak concentration* | NA | NA | NA |
|  |  | *Trough concentration（4th dose）* | 0.75 | 0.81 | 7 |
|  |  | *Peak concentration（4th dose）* | 2.97 | 2.91 | 2 |
| *18* | Initial dose 100 IU; Each dose 100 IU，Q12h;  Infusion time 1h | *First dose peak concentration* | NA | NA | NA |
|  |  | *Trough concentration（5th dose）* | 0.52 | 0.47 | 10 |
|  |  | *Peak concentration（5th dose）* | 4.08 | 4.01 | 2 |
| *19* | Initial dose 150 IU; Each dose 100 IU，Q12h;  Infusion time 1h | *First dose peak concentration* | NA | NA | NA |
|  |  | *Trough concentration（9th dose）* | 0.66 | 0.64 | 3 |
|  |  | *Peak concentration（9th dose）* | 2.11 | 2.06 | 2 |
| *20* | Initial dose 100 IU; Each dose 100 IU，Q12h;  Infusion time 1h | *First dose peak concentration* | NA | NA | NA |
|  |  | *Trough concentration（8th dose）* | 1.42 | 1.28 | 10 |
|  |  | *Peak concentration（8th dose）* | 1.84 | 1.93 | 5 |
| *21* | Initial dose 125 IU; Each dose 75 IU，Q12h;  Infusion time 3h | *First dose peak concentration* | 1.51 | 1.38 | 9 |
|  |  | *Trough concentration（5th dose）* | 0.69 | 0.51 | 26 |
|  |  | *Peak concentration（5th dose）* | 0.97 | 1.17 | 21 |
| *22* | Initial dose 100 IU; Each dose 100 IU，Q12h;  Infusion time 1h | *First dose peak concentration* | NA | NA | NA |
|  |  | *Trough concentration（7th dose）* | 0.64 | 0.61 | 4 |
|  |  | *Peak concentration（7th dose）* | 1.63 | 1.59 | 3 |
| *23* | Initial dose 125 IU; Each dose 75 IU，Q12h;  Infusion time 3h | *First dose peak concentration* | 0.74 | 0.80 | 9 |
|  |  | *Trough concentration（4th dose）* | 0.24 | 0.36 | 47 |
|  |  | *Peak concentration（4th dose）* | 0.85 | 0.82 | 3 |
| *24* | Initial dose 100 IU; Each dose 100 IU，Q12h;  Infusion time 1h | *First dose peak concentration* | NA | NA | NA |
|  |  | *Trough concentration（9th dose）* | 1.16 | 1.15 | 1 |
|  |  | *Peak concentration（9th dose）* | 2.24 | 2.21 | 2 |
| *25* | Initial dose 100 IU; Each dose 100 IU，Q12h;  Infusion time 1h | *First dose peak concentration* | NA | NA | NA |
|  |  | *Trough concentration（21th dose）* | 3.73 | 3.71 | 1 |
|  |  | *Peak concentration（21th dose）* | 4.69 | 4.67 | 0 |
| *26* | Initial dose 150 IU; Each dose 100 IU，Q12h;  Infusion time 1h | *First dose peak concentration* | NA | NA | NA |
|  |  | *Trough concentration（11th dose）* | 0.64 | 0.60 | 6 |
|  |  | *Peak concentration（11th dose）* | 1.25 | 1.29 | 3 |

**Supplementary Table 3 The External validation Predictive Performance of the 26 patients**

| **Sampling scenarios ^a^** | **MSE (%)** | **MAE (%)** | **RMSE (%)** | **MAPE (%)** |
| --- | --- | --- | --- | --- |
| *C_all_* | 0.0062 | 0.0585 | 0.0790 | 1.35 |
| *C_peak_* | 0.0050 | 0.0514 | 0.0706 | 1.86 |
| *C_trough_* | 0.0061 | 0.0603 | 0.0780 | -1.30 |
| *First C_peak_* | 0.0123 | 0.0823 | 0.1108 | 10.19 |
| *C_ss_* | 0.0055 | 0.0557 | 0.0743 | 0.31 |

*^a^ C_all_ represent all plasma concentration, C_peak_ represent peak concentration, C_trough_ represent trough concentration, First C_peak_ represent first dose peak concentration, C_ss_ represent steady-state plasma concentration. MSE mean squared error, MAE mean absolute error, RMSE root mean square error, MAPE mean absolute prediction error.*


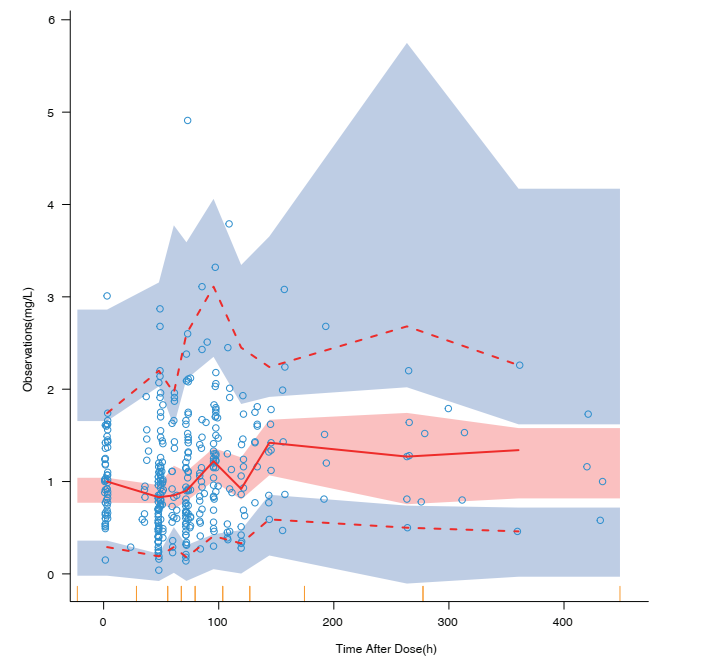


**Supplementary Figure 1.** The prediction-corrected visual predictive check (PC-VPC) of the final model. The red lines represent the 5th, 50th, and 95th percentiles of the observed concentrations; the shaded areas represent the 80% confidence intervals of the 5th, 50th, and 95th percentiles of the simulated concentrations; the dots represent the observed data.
